# Researches on the Pathology and Treatment of Some of the Most Important Diseases of Women

**Published:** 1841-06

**Authors:** 


### REVIEWS.

*Researches on the Pathology and Treatment of some of the most important Diseases of Women:* by ROBERT LEE, M. D., F. R. S., Physician-Accoucheur to the British Lying-in Hospital and the Saint Mary-le-Bone Infirmary; Lecturer on Midwifery in the School of Webb street; London, 1833. pp. 220.

(Concluded from page 304.)

The next disease, which it was the object of Dr. Lee's researches to elucidate, is *phlegmasia dolens,* the pathology of which has been so variously interpreted by different writers. From Mauriceau, who was the first to describe it and who attributed the swelling of the lower extremity to a reflux upon the parts of certain humours which ought to be evacuated by the lochia, through Levret, Puzos, White, Ferriar, Hull, &c. down to Dr. Lee, who thinks he has proved it to be crural phlebitis *commencing in the veins of the uterus,* every variety of hypothesis, which the imagination could suggest, has been framed to explain the phenomena of this singular malady. After all we are not sure that the disease is entirely unveiled, though it must be confessed that the investigations of our author have shed much light upon it. It cannot be doubted that in the cases reported by Dr. Lee, inflammation of the iliac and femoral veins was present and consequently that phlebitis is at least a part of the disease, if it be not the whole or the primitive and essential lesion. That the reader may form his own judgment of the correctness of this statement, we proceed to lay before him the facts and observations collected by our author.

Twenty-two examples of the disease in puerperal women came under the immediate observation of Dr. Lee; in seven of these the disease commenced between the fourth and twelfth days after delivery, and in the remaining fifteen, it appeared subsequent to the end of the second week after parturition. The author shall be allowed to speak for himself as to the observations which he made on these cases:

"In most of the patients there was either an attack of uterine inflammation in the interval between delivery and the commencement of the swelling in the lower extremity, or there were certain symptoms present, which I have before described as characteristic of venous inflammation, viz. rigors, headache, prostration of strength, a small rapid pulse, nausea, loaded tongue, and thirst.

"The sense of pain at first experienced in the uterine region has afterwards been chiefly felt along the brim of the pelvis, in the direction of the iliac veins, and has been succeeded by tension and swelling of the part. After an interval of one or more days, the painful tumefaction of the iliac and inguinal regions, has extended along the course of the crural vessels, under Poupart's ligament, to the upper part of the thigh, and has descended from thence in the direction of the great blood-vessels to the ham. Pressure along the course of the iliac and femoral vessels has never failed to aggravate the pain, and in no other part of the limb has pressure produced much uneasiness. There has generally been a sensible fulness perceptible above Poupart's ligament before any tenderness has been experienced along the course of the femoral vessels; and in every case at the commencement of the attack, I have been able to trace the femoral vein proceeding down the thigh like a hard cord, which rolled under the fingers.

"A considerable swelling of the limb, commencing in the thigh and gradually descending to the ham, has generally taken place in the course of two or three days, and in some cases immediately after the pain has been experienced in the groin. In other cases the swelling has been first observed in the ham or calf of the leg, and has spread from these parts upward and downward until the whole extremity has become greatly enlarged. The integuments have then become tense, elastic, hot, and shining, and in most cases where the swelling has taken place rapidly, there has been no pitting upon pressure, or discolouration of the skin. In several well-marked cases, however, of crural phlebitis at the invasion of the disease, the impression of the finger has remained in different parts of the limb, more particularly along the tibia; but as the intumescence has increased, the pitting upon pressure has disappeared, until the acute stage has passed away. At the onset of the disease I have also observed, in several cases, a diffuse erythematous redness of the integuments along the inner part of the thigh and leg. In one individual only, has suppuration of the glands taken place in the vicinity of the femoral vein; but in several, by an extension of the inflammation, the inguinal glands have become indurated and enlarged. In some women the inflammation of the femoral vein has appeared to be suddenly arrested at the part where the trunk of the saphena enters it, and the inflammation has extended along the superficial veins to the leg and foot. The swelling and pain in these instances have been greatest along the inner surface of the thigh, in the course of the saphena veins. In most cases of crural phlebitis, not only the whole lower extremity, but the nates and vulva, have been affected with a glossy, hot, colourless, and painful swelling, which has not retained the impression of the finger."

The above, which may be considered a very fair description of the acute stage of phlegmasia dolens, points very manifestly to the veins of the affected limb as the seat of morbid action. Evidence of this fact more conclusive still was furnished in the second stage of the malady, when upon the general disappearance of the swelling, first of the thigh and then of the leg and foot, in one case, "large clusters of dilated superficial veins were seen proceeding from the foot, along the leg and thigh, to the trunk; and numerous veins as large as a finger were observed over the lower part of the abdominal parietes."

With the view to illustrate the phenomena of inflammation of the iliac and femoral veins in puerperal women, Dr. Lee relates eight cases which terminated fatally, and in which post mortem examinations were made. One of these cases we quote entire, asking particular attention to the facts revealed by the autopsy.

"Mrs. Edwards, æt. 35, No. 54, King-street, Long Acre, 16th of April, 1829, was delivered of her second child, three weeks ago, after a natural labor, and on the 9th instant was attacked suddenly with pain in the calf of the right leg, and loss of power in the whole right inferior extremity. On the 13th, a considerable swelling, without discolouration, had taken place from the ham to the foot, and great tenderness was experienced along the inner surface of the thigh to the groin. The extremity is now universally swollen, painful, and deprived of all power of motion. The temperature along the inner surface of the limb is increased; the integuments are pale and glistening, and do not pit upon pressure. There is no pain in the hypogastrium, but pressure along the course of the crural vessels excites great suffering, and the vein from the groin to the middle of the thigh is indurated, enlarged, and exquisitely sensible. There is also great sensibility in the ham, and along the inner surface of the leg to the ankle, and where some branches of the superficial veins are hard and painful on pressure. Pulse 80. Tongue much loaded; thirst. Bowels open. It is reported that there was no rigor or symptom of pyrexia, at the invasion of the disease. Twelve years ago, after the birth of her first child, the patient and her relatives state, that she experienced an attack similar to the present in the same limb, and that it remained in a weak condition for several months afterwards, but ultimately recovered its natural size and power.

"18th of April. The tension and increased heat of the limb are somewhat diminished, but the pain continues in the course of the vessels. May 1st. Affection declining. The femoral vein cannot now be felt, but there is still a sense of tenderness in its course down the thigh. No pitting on pressure. She has suffered, during three or four days, considerable uneasiness between the umbilicus and pubis, as well as in the loins, and has had rigors, with quick pulse, loaded tongue, and thirst. The abdomen is soft, but tender on pressure around the umbilicus. 9th. The swelling of the limb is nearly gone, as is the tenderness in the course of the femoral vessels. For several days past, she has experienced attacks of acute pain in the umbilical region, loins and back, which have assumed a regular intermittent form. Every afternoon there has been a violent rigor of an hour's duration, followed by increased heat and profuse perspiration. In the course of the last and preceding nights, there has been slight delirium. The skin is now hot and dry. Pulse 125; tongue brown and parched. Bowels open. The abdomen is neither tense nor swollen. On pressing around the umbilicus, she complains of a deep-seated feeling of soreness. A strong vibratory motion corresponding with the pulsations of the heart, is perceived in the epigastrium.

"21st. The febrile attacks gradually declined in severity, and she appeared to be recovering until yesterday, when she had a long and violent fit of cold shivering. The pulse is extremely rapid and feeble, and the countenance expressive of deep anxiety. There remains no trace of the affection of the lower extremity. 23rd. Has been vomiting at intervals ever since yesterday. Complains of great pain in the left side, increased upon taking a deep inspiration. Pulse 120. 24th. Great prostration of strength and delirium. Surface of the body has assumed a peculiar sallow tinge. The conjunctiva of the right eye has suddenly become of a deep red colour, and so much swollen that the eyelids cannot be closed. The cornea is dull, and she makes little complaint of pain of the eye, and there is no intolerance of light.

"5th. Repeated attacks of vomiting. Debility rapidly increasing; respiration hurried; incessant hacking cough. Pulse 140, feeble; surface of the body cold and clammy. Tongue and teeth covered with dark sordes; diarrhœa. The left eye has also become red and swollen, without much increased sensibility. 26th. Great debility; when undisturbed she is delirious, but seems conscious when roused, and complains of pain in the left side of the chest. Pulse 140. Tongue black and dry. Conjunctiva of left eye also affected with swelling and intense redness. The cornea is dull, and shreds of lymph have been effused over the left iris.

"2d. June. Vision lost. Eyes swollen and pushed forward from the orbits. Great debility. A red puffy swelling has suddenly appeared over the right elbow joint. Diarrhœa. Constant wandering. Hurried and laborious respiration. 15th, died.

"*Inspection.*—Thorax: In its left cavity were contained, upwards of two pints of a thin purulent fluid, and extensive recent adhesions existed between the pleura covering the lower margin of the superior lobe, and the pleura costalis. The substance of this lobe was of a dark colour, approaching to black, and soft in texture, so as to be readily broken down with the fingers. In its centre, about an ounce of cream-coloured pus was found deposited in the dark-coloured and softened lung. This was not contained in a cyst, or membrane, but infiltrated into the pulmonary tissue. In the right cavity of the chest, recent adhesions also existed at the inferior part. A portion of the right inferior lobe was entirely changed from the healthy structure, being converted into a dense, solid, dark-coloured mass. On the anterior surface of this lobe, the pleura was elevated as if by a hard irregular tumor, but when cut into, no pus escaped from this part, and it presented only the appearance of the surrounding portions of lung, with a greater degree of condensation.

"*Vena cava inferior.*—Coats of the vessel considerably thickened, and the internal, where visible, of a scarlet colour; its whole cavity occupied by a coagulum, distending it to its utmost extent, and terminating in a loose pointed extremity, about an inch below the entrance of the vena cava hepatica. The coagulum, covered with a membranous-like investiture of a bright red colour, throughout firmly, and in many places, inseparably adherent to the inner lining of the vein; the substance within it varied in consistence and colour, in some parts it presented the appearance of coagulable lymph; in others, it was a pultaceous dull yellow mass, made up apparently of pus and lymph, blended together. The exterior of the firmer portions were separated into layers, which gradually disappeared as they approached the centre. The mouths of all the veins emptying themselves into the cava were sealed up, the emulgents excepted; the coagulum, near the entrance of these vessels, hanging loosely within the cava.

"*Left common iliac, and its branches.*—Its interior plugged up with a continuation of the coagulum from the cava, and differing in no respect from it, either as to consistence, colour, or the firmness of its adhesions to the inner tunic of the vein; it was continued beyond the entrance of the internal iliac, (which it completely closed,) and terminated in a pointed extremity about the middle of the external iliac; neither the remainder of this vessel, nor the femoral vein, exhibited any morbid changes. The internal iliac was much contracted, and lined with a thick adventitious membrane.

"*Right common iliac, and its branches.*—This vessel was contracted to more than one half of its natural size; it was firm to the touch, and of a greyish blue colour; to its internal coat adhered an adventitious membrane of the same colour, containing within it a firm coagulum, made up of thin layers of dense lymph. The internal iliac was rendered quite impervious by dense dark-coloured bluish membranes, and at its entrance into the common iliac converted into a solid cord.

"The contracted external iliac, contained within it a soft yellowish coagulum, similar to that in the cava; its coats were three or four times their natural thickness, and lined with dark-coloured membranous layers.

"*The femoral vein*, from Poupart's ligament to the middle of the thigh, was diminished in size, and almost inseparable from the artery. Its tunics were thickened, and its interior coated with a dense membrane, surrounding a solid purple coagulum strongly adherent to it. The superficial, and deep femoral veins, were in a similar condition; and the saphena major, and minor, differed from the femoral veins only in the size of the coagulum they contained, which was slender, and had formed no adhesions with the layers of lymph lining their cavity. The cellular membrane, and other textures of the limb, were in a perfectly healthy condition, and in size and appearance, there was externally no visible difference between the two extremities."

In the above case there was the most conclusive evidence of the existence of venous inflammation, to the extent disclosed by the examination; but the uterine veins were not inspected, because at the date of its occurrence Dr. Lee was ignorant of the fact, which his subsequent cases taught him, that they too are implicated, as was proved by their being found with their coats thickened and their calibres contracted and plugged up with coagula and adventitious membranes, from the branches in the uterus to the termination of the hypogastric in the iliac veins. By this discovery Dr. Lee advanced a step further than Dr. Davis, who before him had maintained that phlegmasia dolens depends on inflammation of the iliac and femoral veins, and supported his views by cases and dissections.

Dr. Lee gives several cases of crural phlebitis in women, arising from suppressed menstruation, malignant ulceration of the os and cervix uteri, as well as some other organic diseases of the uterine organs, and also in men, commencing sometimes in the hemorrhoidal or vesical veins, but more frequently from inflammation of the superficial veins of the leg, excited by injuries, ulcers, exposure to cold, &c.; which our limits will not permit us to notice particularly, and we shall close our examination of this part of the work with a brief analysis of the section on the *treatment of crural phlebitis in puerperal women.*

When we reflect upon the evidently inflammatory character of this painful malady, whether, with our author, we believe the inflammation to be seated in the veins, or, with others, in the white lymphatic vessels of the cellular tissue of the limb, we should be led to suppose that bloodletting would be found to be applicable to all cases. In fact Dr. Dewees and others prescribe bleeding unreservedly and direct its repetition, if necessary, until the force of the circulation is subdued and the violence of the pain abated. But Dr. Lee avers that in all the cases he has seen there has been so much feebleness of pulse, and prostration of strength, that he did not venture to draw blood from the arm, but trusted for the relief of the inflammation to the repeated application of leeches above and below Poupart's ligament in the course of the crural veins.

In commencing the treatment of a case of phlegmasia dolens, the judicious practitioner will not be governed by the experience or theoretical views of any one, or any speculative notions of his own, in deciding on the propriety of general bleeding. He will bleed or not, according to the strength and ability of his patient to bear the loss of blood, and the degree and kind of constitutional disturbance that may attend. Should the peculiar train of symptoms be present, which mark the existence of phlebitis, bleeding is not so promising a remedy as in other phlegmasiae, and experience would seem to forbid its employment. If such symptoms are not developed, either because there is no inflammation of the veins or pus has not been poured out to taint the healthful current of the blood, and the attendant fever, pain, &c. demand it, bleeding may be practised with as much freedom as in other inflammatory affections.

Purgatives, in most acute diseases a valuable class of remedies, are peculiarly indicated in phlegmasia dolens, because, as Dr. Lee remarks, the bowels are often much disordered, and with him we entirely agree in preferring repeated small doses of calomel and antimony, because the best effects are obtained from them and they are more generally applicable in puerperal maladies than any other cathartics. Dr. Dewees, who is very high authority in this country, is partial to saline purgatives, and directs drachm doses of Epsom salts and calcined magnesia until they operate freely.

Opium, condemned by many, may be advantageously used after bleeding and purging; indeed, its employment is indispensable to procure rest and relief from pain. The best form for administering it is in the Dover's powder, whose exhibition may be so timed as not to interfere with the operation of the purgative medicines.

With respect to topical applications, some patients derive the greatest relief from warm cataplasms, and others from cold or tepid evaporating lotions, such, for example, as a solution of sugar of lead, vinegar and laudanum. When the acute inflammatory symptoms have passed away, and the limb remains in a weak and œdematous state, advantage may be derived from frictions, gently stimulating liniments, and the flannel roller.

The last subject treated of by Dr. Lee is Uterine Hemorrhage, than which there is none of more importance in the whole range of practice; and our author very properly enters into an enquiry concerning *the connexion of the placenta and fœtal membranes with the uterus, and the process employed by nature for the purpose of suppressing uterine hemorrhage*, in order to obtain a solid foundation for his practical precepts. The celebrated John Hunter described the placenta as consisting of two distinct portions, a fœtal and maternal one, united together, and having connexion with the uterus by means of large arteries, passing into the outer or maternal portion and opening into supposed cells, from whence the blood is taken up by large, wide-mouthed veins and conveyed back to the maternal system. This account, corroborated by his brother, Dr. William Hunter, has been very generally received as correct by subsequent writers and teachers until very recently; but Dr. Lee ventures to impugn it, and with Velpeau and others has succeeded, we think, in demonstrating its untenableness. The placenta is in fact but an appendage of the fœtus, is composed essentially of the ramifications of the umbilical arteries and veins, and has but slight connexion with the uterus, there being no blood-vessels passing between them except nutrient ones of small calibre. As this statement is at variance, if we mistake not, with the prevailing belief of the profession at the present time, we shall fortify it by the lucid description given by our author of what he has repeatedly seen.

"If an incision be made through the parietes of the gravid uterus, where the placenta does not adhere, the membrana decidua will be observed lining the internal surface, and numerous minute blood-vessels and fibres passing from the inner membrane of the uterus to the decidua. At the circumference of the placenta, the membrana decidua separates from the chorion and amnion to pass between the uterus and placenta, and thus forms a complete membranous septum, which is interposed betwixt these organs. The chorion and amnion cover the fœtal surface of the placenta; and between these two membranes and the decidua lie the ramifications of the umbilical vein, and arteries subdivided to an almost indefinite extent, and connected together by white slender filaments running in various directions. The placenta thus consists solely of a congeries of the umbilical vessels, covered on the fœtal surface by the chorion and amnion, and on the uterine surface by the deciduous membrane and enclosed between these membranes; it adheres to the fundus, or some part of the uterus, by innumerable flocculent fibres and vessels.

"On detaching the placenta carefully from the uterus, the deciduous membrane is found to adhere so closely to the umbilical vessels which it covers, that it is impossible to remove it without tearing these vessels. With the fibres uniting the placental decidua to the uterus, are mingled numerous small blood-vessels, proceeding from the inner membrane of the ute rus to the decidua; and these vessels, though more numerous at the connexion of the placenta with the uterus, exist univer sally throughout the whole extent of the membrane. There is no vestige of the passage of any great blood-vessel, either artery or vein, through the intervening decidua, from the uterus to the placenta; nor has the appearance of the orifice of a vessel been discovered, even with the help of a magnifier, on the uterine surface of the placenta. This surface of the placenta, deprived of the deciduous membrane, presents a mass of floating vessels, its texture being exceedingly soft and easily torn; and no cells are discernible in its structure, by the minutest examination."

How then, it may be asked, could the Hunters have been so deceived when they described large vessels passing and repassing between the uterus and placenta? An explanation is to be found in the manner in which their researches were conducted; they did not dissect the gravid uterus in its *natural* state, but after its arteries had been filled with injections of wax forcibly thrown into them. No wonder was it that after this an irregular mass of injected matter should be found between the placenta and uterus, and portions of this same matter contained in the substance of the placenta itself; into these it would readily escape by extravasation. But Mr Hunter found, moreover, on separating this layer of wax interposed between the placenta and uterus, that regular pieces of wax were broken off, which could be traced into the arteries and veins of the uterus. Such are the appearances exhibited by a preparation of the uterus with the placenta adhering to the inner surface in the Museum of the Royal College of Surgeons in London, which is supposed to have been put up by Mr. Hunter himself fifty or sixty years ago. This important preparation, which for half a century has been regarded as affording proof of the cellular structure of the placenta and a communication by great arteries and veins between these and the uterus, Dr. Lee has carefully examined, and he considers it demonstrative of the fact that no such communication exists—the wax having escaped from the orifices of the uterine veins. This singular anomaly in the vascular structure of the uterus we had an opportunity of examining most satisfactorily winter before last, and demonstrating it to the medical class. On the inner surface of the uterus, where the placenta had been recently attached, may be seen a number of smooth, well defined orifices of veins, some of them sufficiently large to admit the tip of the little finger. These orifices, which play an important part in the floodings that sometimes occur in the latter months of gestation, and also during and shortly after labor, are well described by Dr. Lee in the following quotation:

"At that part of the surface of the uterus to which the placenta has been adherent, there are observable a great number of openings leading obliquely through the inner membrane of the uterus, and large enough to admit the point of the little finger: their edges are perfectly smooth, and present not the slightest appearance of having been lacerated by the removal of the placenta. In some places they have a semilunar or elliptical form, and in others they resemble a double valvular aperture. Over these openings in the inner membrane of the uterus, the placenta, covered by deciduous membrane, is directly applied, and closes them in such a manner that the maternal blood, as it flows into the uterine sinuses, cannot possibly escape either into the cavity of the uterus or into the substance of the placenta."

With regard to the natural resources for preventing and arresting uterine hemorrhage, on the promotion of which our hopes of relieving this formidable accident must depend, Dr. Lee holds the following language, which, as it accurately embodies our own views, we give without comment.

"After the separation of the placenta in natural labour, the contractions of the uterus, and the formation of clots within its cavity, and in the orifices of the uterine sinuses, are the principal means employed by nature for arresting the flow of blood. The semilunar or valvular-like edges of the vessels at their termination in the inner surface of the uterus, are admirably adapted to ensure the effect of arresting the current of blood through these passages by the contraction of the fibres with which they are every where surrounded. However excited the circulation in the uterine vessels may be, the structure of the parts is such, that flooding cannot take place from a contracted uterus after the expulsion of its contents. All the different means which have been hitherto recommended for checking the effusion of blood in uterine hemorrhage produce their effect either by exciting contraction of the uterus, and the subsequent closure of the bleeding orifices, or by promoting the coagulation of the blood itself within them."

Our author devotes separate chapters to the consideration of hemorrhage occurring during pregnancy, according as the placenta may be attached over the os uteri or to the fundus and body of the organ,—recognizing to the fullest extent the important practical distinction first made by Levret in France, and since more generally known to English and American practitioners through the writings of Mr. Rigby of Norwich. In the first case, towards the close of pregnancy, hemorrhage is *inevitable* from the expansion of the cervix uteri, while in the second it is entirely casual or accidental: in both, however, the effusion of blood is owing to the separation of the placenta, to a greater or less extent, and the consequent exposure of some of the large venous openings that have been described. In both, also, the hemorrhage being purely passive can only be arrested by a power extraneous to the vessels themselves, and this power resides in the contraction of the muscular fibres of the uterus. Here, however, the parallel ceases; when the placenta is attached over the os uteri, the contractions of the organ tend immediately to increase the hemorrhage by breaking up still further the attachments of the placenta, and hence the increased flow of blood during the pains; on the other hand, when the placenta is attached to a higher portion of the uterus, the immediate effect of muscular contraction is to diminish the discharge or to intercept it altogether, provided it can be efficiently exerted. On this difference is founded the different treatment which these two kinds of hemorrhage exact; artificial delivery being the remedy for the inevitable hemorrhage, while rupturing the membranes to allow the organ to contract more powerfully is the remedy for the accidental.

With regard to delivery as a means of relieviug hemorrhage of the first species, the conditions necessary to insure the success or even the safety of the operation should never be lost sight of, viz., a soft and dilatable state of the os uteri, and until this takes place our author very properly recommends the recumbent postute, cold applications to the hypogastrium and pubis, and the introduction of a large piece of soft sponge within the vagina, as the remedies on which the greatest reliance is to be placed, until delivery is safely practicable. Against the last of these measures, Dr. Meigs objects in his excellent manual, the Philadelphia Practice of Midwifery, and we took the opportunity afforded by a notice of his work in a former number of this Journal, to express our dissent from the views inculcated by him. What was said on that occasion need not be repeated; we are thoroughly satisfied that the tampon is the most valuable resource which can be commanded in these critical cases, but it must be employed with judgment. Dr. Lee, for example, very pointedly and properly forbids the tampon when the os uteri is soft and yielding, because then it is no longer to be relied on and is no security against dangerous, concealed hemorrhage, while he commends it as a valuable remedy in rigid undilatable states of the os uteri, to command the flow of blood until the proper time for delivery arrives.

The treatment of accidental hemorrhage has already been indicated in general terms; Dr. Lee describes it more particularly in the following extract:

"Although there should be little or no disposition in the uterus to contract and expel the child, if the flooding continues and the strength of the woman is obviously much impaired by it, the fœtal membranes should be ruptured, the liquor amnii evacuated, and the uterus roused to contraction by friction over the hypogastrium, and the dilatation of the os and cervix uteri by two fingers introduced within them. If there are labor pains there will be little difficulty in tearing the membranes with the point of the finger when they are tense; if there are no pains the best mode of perforating the membranes is to introduce a slender silver catheter through the mouth of the womb, and carry it forward till the membranes are pierced and the liquor amnii begins to escape. I have seen more than one fatal case of uterine hemorrhage, where the practice of Puzos was adopted, but I am convinced, had the membranes been ruptured before the powers of the system and of the uterus had been less impaired, the result would have been different."

Here we have a correct enumeration of the several operative procedures which are so well known and generally employed since the time of Puzos: but we regret to observe, nevertheless, a departure from the *method* of that distinguished and original writer, which, if we mistake not, has led to disappointment and even fatal results in the hands of Ramsbotham and others. According to our author the membranes are first to be ruptured and then the uterus is to be roused to contraction, thus reversing the order of Puzos, who excited the uterine contractions first by stimulating the os uteri with the finger, and then ruptured the membranes, as soon as they are observed to become tense during the pains. In this manner uterine contraction, the great desideratum, is insured, without which the evacuation of the liquor amnii is worse than useless, because, in the flaccid condition of the organ which immediately ensues and may continue for hours or days, blood may be poured out in greater abundance than before, and the practitioner be driven to deliver at last in an exhausted condition of his patient. Such were precisely the circumstances attending some of Ramsbotham's most instructive cases, while in the hands of Rigby, Puzos's method, closely pursued, was infallible. H. M.

